# Enlarged Memory T-Cell Pool and Enhanced Th1-Type Responses in Chronic Myeloid Leukemia Patients Who Have Successfully Discontinued IFN-α Monotherapy

**DOI:** 10.1371/journal.pone.0087794

**Published:** 2014-01-31

**Authors:** Mette Ilander, Anna Kreutzman, Peter Rohon, Teresa Melo, Edgar Faber, Kimmo Porkka, Jukka Vakkila, Satu Mustjoki

**Affiliations:** 1 Hematology Research Unit Helsinki, Department of Medicine, University of Helsinki and Helsinki University Central Hospital, Helsinki, Finland; 2 Department of Hemato-Oncology, Faculty Hospital Olomouc and Faculty of Medicine and Dentistry, Palacky University, Olomouc, Czech Republic; 3 Hospital de S. Joao, Porto, Portugal; University of Thessaly, Greece

## Abstract

A small proportion of chronic myeloid leukemia patients treated with interferon-α (IFN-α) monotherapy are able to discontinue the treatment without disease relapse although residual leukemia cells are present. Recently, we showed that these patients have increased amount of NK-cells and a distinct blood cytokine profile. We now aimed to study the function of NK- and T-cells in order to understand the role of the immune system in maintaining the treatment response after IFN-α discontinuation. The study included 13 patients: 5 patients were still treated with IFN-α monotherapy (IFN-ON, median treatment time 163 months) and 8 had stopped the treatment successfully (IFN-OFF, median time without therapy 42 months). Detailed immunophenotype and cytokine production of NK- and T-cells was analyzed with flow cytometry. In addition, the cytotoxicity of NK-cells was studied using K562 as target cells and both the degranulation and direct killing was measured. Compared to healthy controls, IFN-OFF patients had increased proportion of CD4^+^ effector memory (CCR7^−^CD45RA^−^; median 23% vs. healthy 16%, p = 0.009) and CD8^+^ central memory T-cells (CCR7^+^CD45RA^−^; median 26% vs. healthy 14%, p = 0.004). Further, upon stimulation the IFN-γ/TNF-α cytokine secretion by CD4^+^ T-cells was significantly enhanced in IFN-OFF patients (median 13.7% vs. healthy 7.8%, p = 0.01), and CD4+ effector and central memory cells were the main cytokine producers. No similar increase was observed in IFN-ON group (6.5%). In addition, the proportion of NK-cells was significantly increased in IFN-OFF patients (median IFN-OFF 24%, healthy 13%, p = 0.04), but their direct killing of K562 cells was impaired. The cytotoxicity of NK-cells was also diminished in IFN-ON patients. To conclude, in addition to elevated NK-cell count, IFN-OFF patients have increased amount of memory T-cells, which are able to induce strong cytokine response upon stimulation. This activity may contribute to the maintenance of prolonged remission after successful IFN-α discontinuation.

## Introduction

Chronic myeloid leukemia (CML) is a relatively rare myeloproliferative disorder with an annual incidence of 1–2 cases per 100 000 individuals [1]. It is most often diagnosed in elderly patients with the median age of 65 years. The pathogenesis of the disease is well known and the leukemic transformation is caused by a translocation (9;22) in hematopoietic stem cells (HSCs). This results in a constantly active tyrosine kinase BCR-ABL, which in turn causes unregulated proliferation of hematopoietic cells [2].

Tyrosine kinase inhibitors (TKIs; imatinib, dasatinib, nilotinib) are the current first-line treatment in CML and they have improved the prognosis significantly [3]–[5]. Before the TKI era, CML patients were treated with interferon-α (IFN-α) [6], but only a small proportion of patients responded well to the treatment. However, surprisingly up to half of the patients who had achieved complete cytogenetic remission (CCyR) were able to discontinue the treatment without disease relapse [7], [8]. Despite the increasing understanding of the beneficial effects of IFN-α treatment, it is still unclear why some CML patients are able to stop IFN-α treatment and stay in remission without treatment. It is worth noticing that these patients still have residual leukemic cells left but for unknown reason they do not expand [9], [10]. Therefore, it is conceivable that IFN-α therapy has induced changes in the immune system, which have a protective role. Supporting this theory, several studies have reported that IFN-α induces specific immune response against CML cells [11]–[14]. Due to these encouraging results, several recent clinical trials aiming in the cure of CML have combined IFN-α with TKI therapy [15], [16]. Markedly, the combination therapy has induced more rapid and deeper treatment responses than TKI therapy alone [17]. Furthermore, adding IFN-α to imatinib-treatment seems to increase the possibility to discontinue the treatment successfully [18], [19].

Because of the comeback of IFN-α in the treatment of CML, it is even more important to understand the immunomodulatory mechanisms induced by the drug. Our group has previously shown that IFN-α treated CML patients who have successfully discontinued the treatment have increased amounts of NK-cells and CD8^+^ T-cells, and a distinct cytokine profile [20]. To better understand the role of NK- and T-cells in the putative curative action of IFN-α, we now aimed to study their function and phenotype in more detail, and analyzed primary samples from CML patients who have successfully discontinued IFN-α monotherapy without disease relapse.

## Patients and Methods

### Study Patients and Samples

The study population included 13 chronic phase CML patients treated with IFN-α monotherapy (Table 1) and no TKI treatment has been used in these patients. 5 patients were currently treated with IFN-α monotherapy (IFN-ON) and 8 had stopped the treatment successfully (IFN-OFF). Two of the IFN-ON patients were pregnant at the time of sample withdrawal and they are marked with separate dots in the graphs. Samples from 14 age and sex matched healthy volunteers were used as controls. The mean age of the healthy controls was 55 years, and in the patient cohort it was 56 years at the time of sample collection. The patient number was limited in this study as TKIs are the current first-line treatment in CML, and thus IFN-α monotherapy treated patients are very rare. We therefore extended the patient sample collection to 3 different countries (Finland, Czech Republic and Portugal).

**Table 1:** Patient characteristics.

IFN status	Dg	Gender	Ageat dg.	Duration of CML (months)	Reason for STOP/no STOP	Course of IFN-α therapy	Response
IFN-ON 1	CP	m	62	144	No severe side-effects,PCR positive	auto-PBSCT, 1.5 MU/2x weekly – cont.For 12 years	MR4.0
IFN-ON 2	CP	f	35	240	No severe side effects,	2 MU/3x weekly – cont. for 20 years.	CMR
					doctor hesitates to stop,		
					sometimes PCR positive		
IFN-ON 3	CP	m	29	163	Patient does not want to stop	5.5 MU/6x weekly – cont. for 14 years.	MR4.0
IFN-ON 4	CP	f*	20	168	no severe side-effects	busulfanc, 1.5 MU/2x weekly	MMR
					PCR positive	– cont. for 12 years.	
IFN-ON 5	CP	f*	40	8	Diagnosed during pregnancy	For 8 months	CMR
IFN-OFF 1	CP	m	44	204	Nephrosis,	IFN-α (10 years and stopped),	CMR
					low hemoglobin (80)	7 years no therapy	
IFN-OFF 2	CP	m	53	170	Side-effects(nausea, muscle pain),	IFN-α (11 years and stopped),	CMR
					good response,patient requested	3 years no therapy	
IFN-OFF 3	CP	f	53	134	Good response	auto-PBSCT, IFN-α (8 years and stopped),	CMR
						3 years no therapy	
IFN-OFF 4	CP	m	59	86	Good response	IFN-α (5 years and stopped),	CMR
						2 years no therapy	
IFN-OFF 5	CP	m	42	84	Good response	IFN-α (5 years and stopped),	CMR
						3 years no therapy	
IFN-OFF 6	CP	m	36	112	Good response	auto-PBSCT, IFN-α (5 years and stopped),	CMR
						4 years no therapy	
IFN-OFF 7	CP	f	41	172	Good response	auto-PBSCT, IFN-α (7 years and stopped),	CMR
						7 years no therapy	
IFN-OFF 8	CP	f	54	168	Good response	IFN-α (10 years and stopped), 4 years no therapy	CMR

* = Pregnant. Abbreviations: CP, chronic phase; m, male; f, female; dg, diagnosis; cont, continued; MU, million unit; MR4.0, molecular response 4.0, CMR, Complete Molecular Response; MMR, Major Molecular Response; Auto-PBSCT, autologous peripheral blood stem cell transplantation.

The abbreviation IFN-ON is used for patients who were treated with IFN-α at the time of sampling and IFN-OFF for patients who have been able to stop the IFN-α treatment and have no ongoing treatment for CML.

Peripheral blood (PB) samples were collected from all patients and healthy controls. Mononuclear cells (MNCs) were separated by Ficoll gradient centrifugation (GE healthcare, Buckinghamshire, UK) and stored at liquid nitrogen or analyzed fresh.

### Ethics Statement

All patients and healthy controls gave their written informed consent and the study was approved by Helsinki University Central Hospital (Helsinki, Finland), University Hospital Olomouc (Olomouc, Czech Republic) and S. Joao University Hospital (Porto, Portugal) ethics committees. The study was conducted in accordance with the Declaration of Helsinki.

### T-cell Phenotyping

Thawed MNCs were stained with anti-CD45-APC-H7 (clone 2D1), -CD3-PeCy7 (SK7), -CD4-PerCP (SK3), -CD45RA-AlexaFluor700 (GB11) and -CCR7-PE (150503) (R&D Systems, Minneapolis, MN, USA) antibodies. The stained MNCs were acquired with FACSAriaII and analysed with FlowJo (Version 9.6.1, TreeStar). All antibodies were purchased from BD Biosciences unless mentioned otherwise.

### Activation of T-cells

Thawed MNCs were stimulated with anti-CD3 (clone OKT3, 2,5 µg/ml) and co-stimulatory anti-CD28 (L293, 1 µg/ml) and anti-CD49d (L25, 0.5 µg/ml) (BD biosciences) and incubated for 6 hours at +37°C. MNCs without stimulation were used as controls. GolgiStop (BD biosciences) was added to each well. After the incubation, the cells were stained with the following antibodies: CD45-APC-H7 (2D1), CD3-APC (SK7), CD4-PerCP (SK3), CD8PE-Cy7 (SK1) and TCRγδ-PE (11F2). After, the cells were permeabilized and fixed according to the Cytofix Cytoperm (BD biosciences, San Diego, CA, USA) kit’s protocol. Intracellular staining was done with Granzyme B-Alexa Fluor 700 (GB11), IFN-γ-FITC (B27) and TNF-α-FITC (MAb11), and 50 000 CD45^+^ cells were analyzed with FACSAria. When the cytokine secretion of CD4^+^ T-cell subsets was analyzed, TCRγδ antibody was replaced with CCR7-PE antibody, and CD45RA-AlexaFluor700 was added to the panel.

### T-cell Degranulation Assay

Thawed MNCs were stimulated with anti-CD3 (clone OKT3, 2,5 µg/ml) and co-stimulatory anti-CD28 (L293, 1 µg/ml) and anti-CD49d (L25, 0.5 µg/ml) (BD biosciences) and incubated for 6 hours at +37°C. The cells were incubated for 6 hours in the presence of antibodies for the degranulation marker CD107a-FITC (H4A3) and CD107b-FITC (H4B4). MNCs without stimulation were used as controls. After the incubation, the cells were stained with the following antibodies: CD45-APC-H7 (2D1), CD3-APC (SK7), CD4-PerCP (SK3), CD8PE-Cy7 (SK1), CCR7-PE (11F2), CD45RA-AlexaFluor700 (GB11). 50 000 CD45^+^ cells were analyzed with FACSAria.

### Immunophenotyping of NK-cells

Freshly isolated MNCs were stained with CD45-APC-H7, CD3-PE-Cy7, CD14-and CD19-Pacific Blue (clones TüK4 and SJ25-C1, Invitrogen), CD56-PE (NCAM16.2), CD16-PE-TexasRed (3G8, Invitrogen), CD57-FITC (NK-1), CD62L-APC (DREG-56), CD27-V500 (M-T271) and CD45RA-AlexaFluor700 (HI100). 50 000 CD45^+^ cells were acquired with FACSAria (BD Biosciences, San Diego, CA, USA) and analyzed with FlowJo.

### Cytotoxicity Assay

The target cell line K562 (Sigma-Aldrich, St. Louis, MO, USA) was stained with CellTrace Violet Cell Proliferation (Invitrogen, Carlsbad, CA, USA) according to the manufacturer’s instructions and rested over night at +37°C.

The next day, the proportion of NK-cells (CD3negCD16/56+) from freshly isolated MNCs was measured by flow cytometry and co-incubated with K562 cells in NK-cell:target ratios of 4∶1 and 8∶1 for 6 h at +37°C. All reactions were done in triplicates. After 6 hours, the cells were stained with Calcein-AM (stains only living cells) from LIVE/DEAD® Viability/Cytotoxicity Kit (Invitrogen) according to the manufactureŕs instructions. Before cytometric analysis, CountBright beads (Invitrogen) were added to each tube and 5000 beads were gated and counted. The gate was set on K562 cells (CellTrace positive) and the live K562 targets (Calcein-AM positive) were calculated.

### NK-cell Degranulation Assay

Freshly isolated MNCs were stimulated with K562 cells at ratio 10∶1. MNCs without targets were used as negative controls. The cells were incubated for 6 hours in the presence of antibodies for the degranulation marker CD107a-FITC (H4A3) and CD107b-FITC (H4B4). The cells were stained with CD45-APC-H7, CD3-APC, CD16-PE-Cy7 (3G8) and CD56-PE. 50 000 CD45^+^ cells were acquired with FACSAria and analyzed with FlowJo.

### NK-cell Cytokine Assay

Fresh MNCs were stimulated with K562 in the ratio 10∶1 or with 2 nM PMA (Cell Signaling Technology, Beverly, MA, USA) and 0.02 µg/µl of Calcium Ionophore (Sigma-Aldrich, Saint Louis, MO, USA). GolgiStop (BD) was added to each well. After 6 h incubation at +37°C, the cells were stained with CD16-PE-Cy7, CD56-PE, CD45-APC-H7 and CD3-APC. The cells were then permeabilized and fixed according to the Cytofix Cytoperm kit’s protocol (BD). Intracellular cytokines IFN-γ and TNF-α were stained with anti-IFN-γ- and anti-TNF-α-FITC and 50 000 CD45^+^ cells were acquired with FACSAria and analyzed with FlowJo.

### Statistical Analysis

All statistical analyses were performed with GraphPad Prism (GraphPad Software Inc., CA, USA). Nonparametric Mann-Whitney test was used for comparison between two groups and two-way Anova for comparison in the cytotoxicity assay. P<0.05 was considered statistically significant.

## Results

### CML Patients Who have been Able to Stop IFN-α Treatment have Increased Amount of Memory CD4+ and CD8+ T-cells

**Figure 1:**
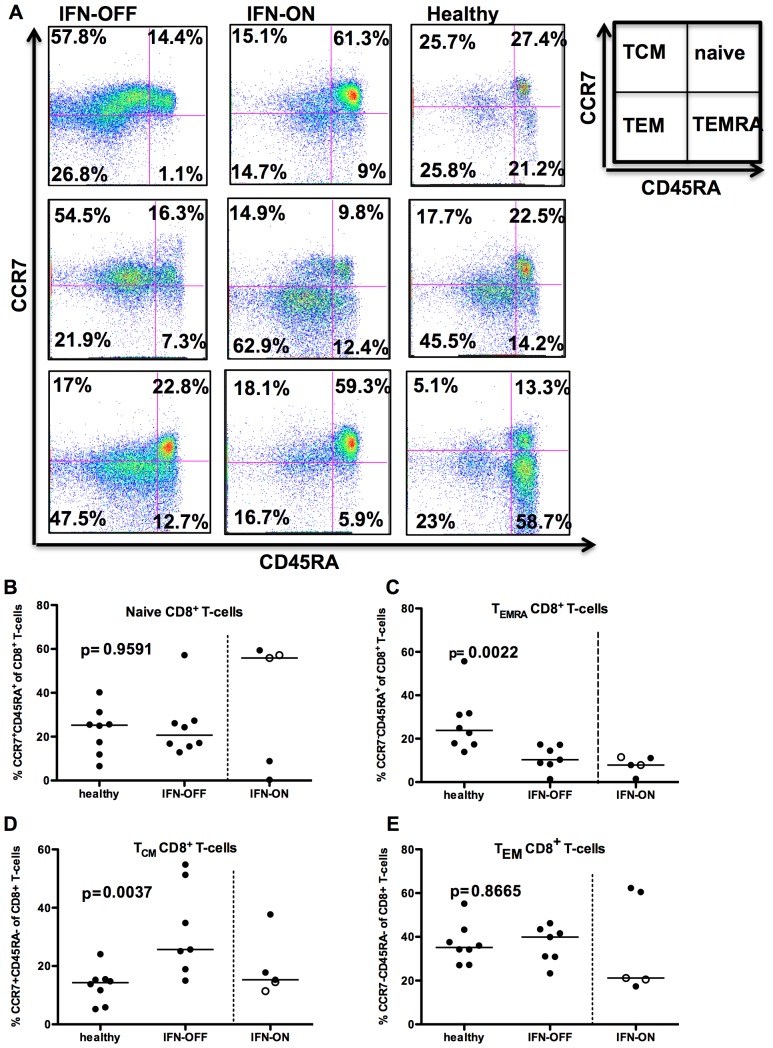
CD8^+^ T-cells from IFN-OFF patients have an increased central memory compartment. Memory status of CD8^+^ T-cells was determined by FACS-analysis using anti-CD45RA and –CCR7 antibodies. **A)** Examples of 3 IFN-OFF, 3 IFN-ON and 3 healthy controls. T_CM_, T central memory cells; T_EM_, T effector memory cells; T_EMRA_, T CD45RA+ effector memory cells **B)** The proportion of naïve CD8^+^ T-cells (CD45RA^+^CCR7^+^), **C)** CD8^+^ CD45RA^+^ effector memory T-cells (CD45RA^+^CCR7^−^), **D)** CD8^+^ central memory T-cells (CD45RA^−^CCR7^+^), and **E)** CD8^+^ effector memory T-cells (CD45RA^−^CCR7^−^) from all CD8+ T-cells. White dots represent pregnant patients in IFN-ON group (Table 1).

To understand the nature and function of T-cells in IFN-α treated CML patients, PB MNCs were phenotyped with memory markers such as CCR7 and CD45RA. In IFN-OFF patients, the proportion of central memory CD8+ cells (CCR7^+^CD45RA^−^) was significantly increased when compared to healthy controls (median IFN-OFF 25.7% vs. healthy 14.3% of CD8^+^ T-cells, p = 0.0037, Figures 1A and 1D). No significant differences were observed in the CD8^+^ effector memory cells (median IFN-OFF 39.9%, healthy 35.2%, p = 0.87, Figure 1E) or naïve CD8+ T-cells (CCR7+CD45RA+) (median IFN-OFF 20.7% vs. healthy 25.3% of CD8^+^ T-cells, p = 0.95, Figures 1A and 1B). However, both patient groups seemed to have a decreased proportion of CD8^+^ CD45RA^+^ effector memory T-cells (T_EMRA_) when compared to the healthy (median IFN-OFF 10.3%, IFN-ON 7.9%, healthy 23.8%, p = 0.002, Figure 1C).

**Figure 2:**
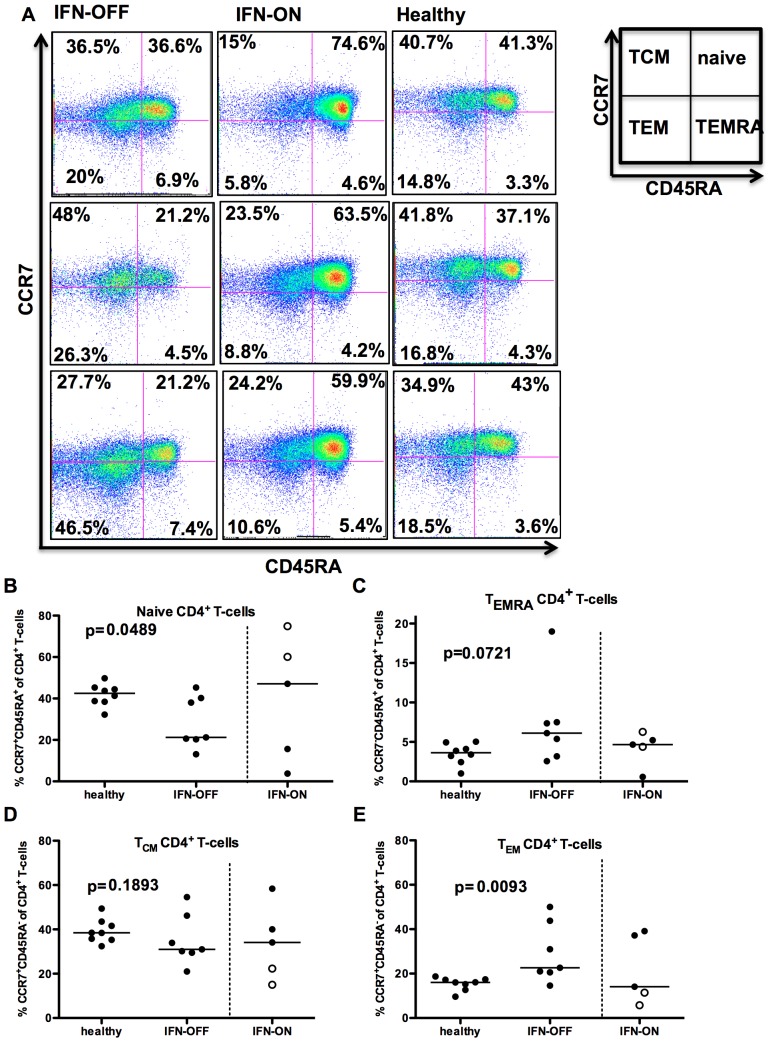
CD4^+^ T-cells from IFN-OFF patients have an increased effector memory compartment. Memory status of CD4^+^ T-cells was determined by FACS-analysis using anti-CD45RA and –CCR7 antibodies. **A)** Examples of 3 IFN-OFF, 3 IFN-ON and 3 healthy controls. T_CM_, T central memory cells; T_EM_, T effector memory cells; T_EMRA_, T CD45RA+ effector memory cells. **B)** The proportion of naïve CD4^+^ T-cells (CD45RA^+^CCR7^+^), **C)** CD45RA^+^ effector memory CD4^+^ T-cells (CD45^+^CCR7^−^), **D)** CD4^+^ central memory T-cells (CD45RA^−^CCR7^+^), and **E)** CD4^+^ effector memory T-cells from all CD8+ T-cells. White dots represent pregnant patients in IFN-ON group (Table 1).

In contrast to the CD8^+^ T-cell compartment, the effector memory (CCR7^−^CD45RA^−^) T-cell pool was significantly enlarged in IFN-OFF patients compared to healthy controls (median IFN-OFF 22.6% vs. healthy 16.1% of CD4^+^ T-cells, p = 0.009, Figures 2A and 2E). The IFN-OFF group also seemed to have an increased proportion of CD4^+^ CD45RA^+^ effector memory T-cells (T_EMRA_) when compared to the healthy (median IFN-OFF 6.1%, healthy 3.6%, p = 0.07, Figure 2C). No differences were observed in the central memory (CCR7^+^CD45RA^−^) subset (Figure 2D), but IFN-OFF patients had a lower frequency of naïve CD4^+^ T-cells (median IFN-OFF 21.2% vs. healthy 42.5% of CD4^+^ T-cells, p = 0.049, Figures 2A and 2B).

In IFN-ON group, the division of different T-cell populations (especially the proportion of naïve CD4^+^ and CD8^+^ cells) varied significantly between individual patients, which could be due to the heterogeneous clinical situation (two patients were pregnant and they are marked with separate dots in Figures 1 and 2).

### Cytokine Secretion by CD4+ T-cells is Significantly Increased in IFN-OFF Group

**Figure 3:**
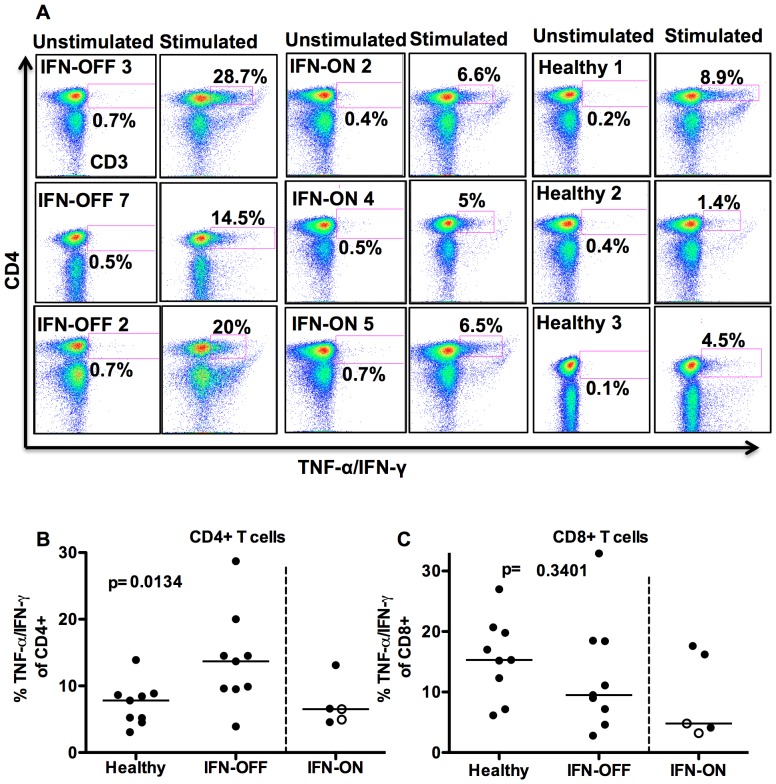
CD4^+^ T-cells from IFN-OFF patients are more prone to secrete TNF-α/IFN-γ than CD4^+^ T-cells from the healthy. T-cells were stimulated with anti-CD3, –CD28 and -CD49d. After 6 h incubation, cell surface markers and intracellular cytokines were stained and analyzed with flow cytometry. **A)** Non-stimulated and stimulated TNF-α/IFN-γ secretion of CD3+ T-cells. Shown are 3 representative patients from each group (IFN-OFF, IFN-ON and healthy). Percentages are calculated from CD4^+^ T-cell population. **B)** TNF-α/IFN-γ secretion of CD4^+^ T-cells in all patients **C)** TNF-α/IFN-γ secretion of CD8^+^ T-cells in all patients. White dots represent pregnant patients in IFN-ON group (Table 1).

To study the activation and cytokine secretion potential of T-cells, MNCs were stimulated with anti-CD3, anti-CD28 and anti-CD49d, and the production of IFN-γ and TNF-α was measured by flow cytometry. CD4^+^ T-cells were more active to secrete cytokines in IFN-OFF patients than in healthy controls and significantly higher proportion of cells responded by TNF-α/IFN-γ secretion (IFN-OFF median 13.7%, IFN-ON 6.5%, healthy 7.8%, p = 0.01, Figures 3A and 3B). The cytokine secretion of CD8^+^ T-cells was similar in IFN-α treated CML patients and in healthy controls (IFN-OFF 9.5%, IFN-ON 4.8%, healthy 15.3%, p = 0.3, Figure 3C).

**Figure 4:**
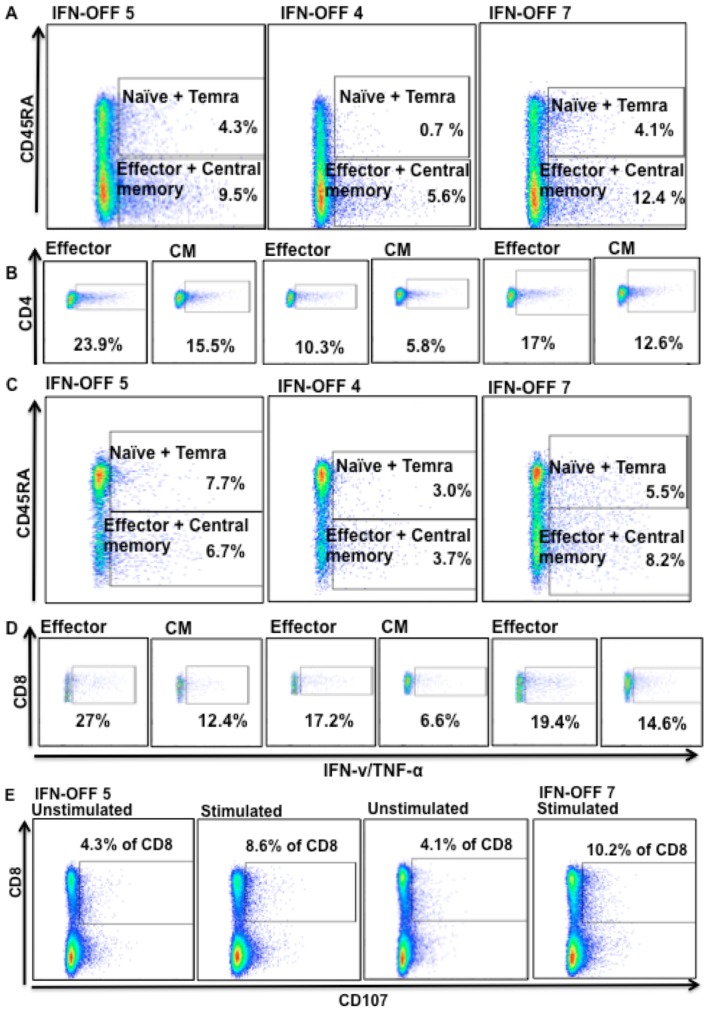
Function of T-cell subsets in IFN-OFF patients. **A)** T-cells were stimulated with anti-CD3, –CD28 and -CD49d and after 6 h incubation, cell surface markers and intracellular cytokines were stained and analyzed with flow cytometry. The cytokine secretion of CD4+ T-cell subsets (effector and central memory, naïve and temra cells) was analyzed separately. The percentage values present the proportion of cytokine secreting cells from all CD4+ cells showing that the effector and central memory T-cells are the main cytokine producers. **B)** The proportion of cytokine secreting effector and central memory CD4^+^ T-cells were also analyzed separately and the % values show the proportion of positive cells from a subset in question. **C)** Cytokine secretion of CD8^+^ T-cell subsets and **D)** the proportion of cytokine secreting effector and central memory CD8^+^ T-cells separately. **E)** Degranulation of CD8^+^ T-cells was analyzed by CD107a/b expression with flow cytometry after 6 h incubation with anti-CD3, -CD28 and -CD49d. Unstimulated MNCs were used as a control.

In addition, we analyzed the cytokine secretion of distinct CD4^+^ and CD8^+^ T-cell memory subsets from 3 IFN-OFF patients. In the CD4^+^ cells, effector memory and central memory CD4^+^ T-cells were clearly most active cell subsets in cytokine secretion (more than 80% of all cytokines secreted by CD4^+^ cells came from effector memory and central memory T-cells) (Figure 4A). In CD8^+^ compartment both naïve and memory cells secreted cytokines, but interestingly the CD8^+^ effector cells responded especially well to stimulation by secreting cytokines (Figure 4C). Degranulation responses were also evaluated from 2 patients from whom suitable samples were available, and the proportion of CD107 expressing increased after the stimulation (Figure 4E). However, due to low number of patients studied, no further conclusions can be withdrawn. The proportion of potentially cytotoxic Granzyme B^+^ T-cells was similar in IFN-OFF patients and healthy controls (data not shown).

### IFN-OFF Group has Increased Proportion of NK-cells Displaying a Mature Phenotype

**Figure 5:**
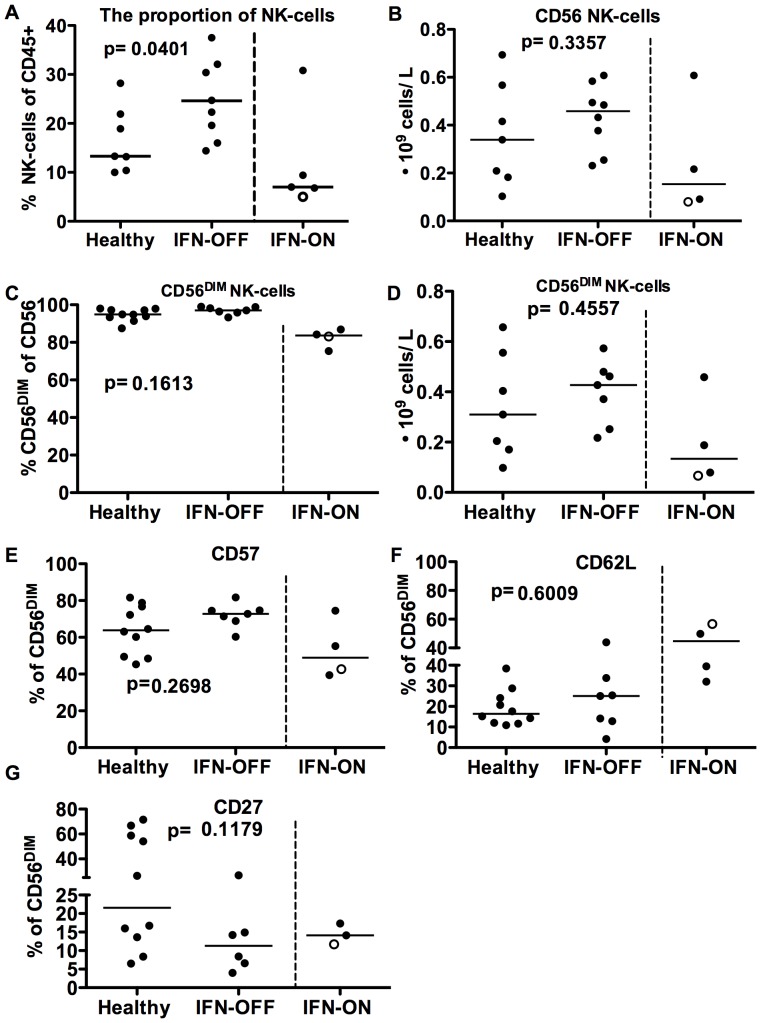
NK-cells from IFN-OFF patients have a mature phenotype. NK-cell proportions and surface markers CD62L, CD57 and CD27 were analyzed with flow cytometry and absolute NK-cell counts were counted from total lymphocyte numbers. **A)** The proportion of NK-cells in IFN-OFF and IFN-ON patients **B)** Absolute amount of NK-cells in IFN-ON and IFN-OFF patients **C)** The proportion of CD56^DIM^ NK-cells from CD56 NK-cells in IFN-ON and IFN-OFF patients **D)** Absolute amounts of CD56^DIM^ NK-cells in IFN-ON and IFN-OFF patients. **E)** CD57 expression in CD56^DIM^ NK-cells. **F)** CD62L expression in CD56^DIM^ NK-cells **G)** CD27 expression in CD56^DIM^ NK-cells. White dots represent pregnant patients in IFN-ON group (Table 1).

Similarly as shown previously [20], the proportion of NK-cells from lymphocytes was significantly increased in IFN-OFF patients (median IFN-OFF 24%, healthy 13%, p = 0.04, Figure 5A), while there was no significant difference in the IFN-ON group (7%). In the absolute NK-cell number the differences between the groups did not reach statistical significance (IFN-OFF median 0.29⋅10^9^ cells/L, healthy 0.18⋅10^9^ cells/L, p = 0.3, Figure 5B). The majority of NK-cells in IFN-OFF patients were CD56^DIM^ cells (median IFN-OFF 97%, healthy 95%, p = 0.16, Figures 5C and D). NK-cells from IFN-ON patients included significantly less CD56^DIM^ cells than NK-cells from IFN-OFF or healthy controls (83.7%, 0.13⋅10^9^ cells/L). Furthermore, the CD57 expression (marker for mature phenotype) in CD56^DIM^ NK-cells seemed to be higher in IFN-OFF group (median IFN-OFF 72.8%, healthy 63.8%, p = 0.27, Figure 5E) and lower in IFN-ON group (49%), while the CD62L (adhesion molecule) expression was increased in IFN-ON patients (Figure 5F). A trend towards lower CD27 expression in NK-cells was also observed in IFN-OFF group (Figure 4G), but no statistically significant differences existed. To conclude, the majority of NK-cells in IFN-OFF patients had a mature CD56^DIM^CD57^+^ phenotype lacking the expression of CD62L and CD27.

### NK-cells from IFN-OFF Patients have Decreased Cytotoxic Capacity

**Figure 6:**
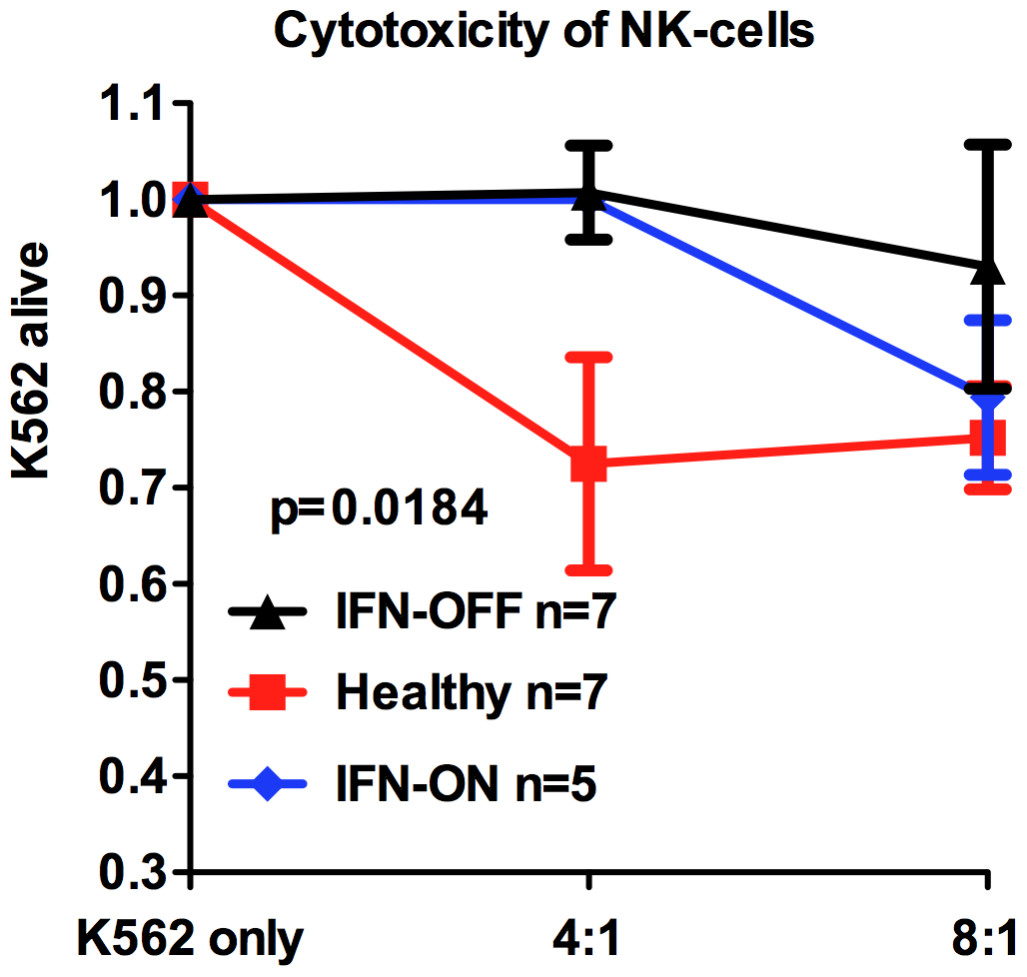
The cytotoxicity of NK-cells from IFN-OFF and IFN-ON patients is impaired. MNCs were used as effector cells and K562 cells as target cells. The NK-cell percentage was determined by flow cytometry and the number of effector MNCs was counted accordingly. Cells were co-incubated for 6 h at +37°C at effector:target ratios 4∶1 and 8∶1. The graphs present alive K562 cells after the co-incubation with effector cells.

To analyze the function of the increased number of NK-cells in IFN-OFF patients, we first measured their cytotoxicity by using MNCs as effector cells and NK-cell susceptible cell-line (K562) as target cells (effector: target ratio was standardized based on NK-cell percentage). Surprisingly, in the both of the patient groups the killing was decreased (median IFN-OFF 1% at ratio 4∶1, IFN-ON 1%) when compared to healthy controls (17%) (Figure 6). From three patients (2 IFN-OFF and 1 IFN-ON) the NK-cell killing assay was concomitantly done with purified NK-cells and MNCs and the results were concordant confirming the impaired NK-cell cytotoxicity.

### The Degranulation of NK-cells from IFN Patients is Similar as in Healthy Controls

**Figure 7:**
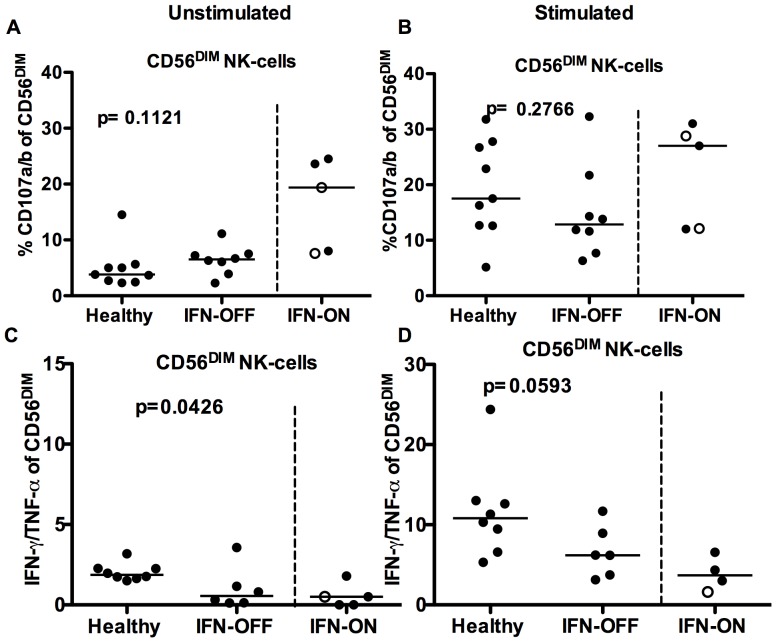
Degranulation and cytokine secretion of NK-cells. MNCs were incubated for 6 hours with and without K562 cells at +37°C and the degranulation (CD107a/b expression) and cytokine secretion of NK-cells was measured by flow cytometry. **A)** Degranulation of NK-cells without stimulation **B)** Degranulation of NK-cells after the stimulation with K562-cells **C)** IFN-γ/TNF-α secretion by NK-cells without stimulation **D)** IFN-γ/TNF-α secretion by NK-cells after the stimulation with K562-cells. White dots represent pregnant patients in IFN-ON group (Table 1).

To further determine the function of NK-cells in IFN-OFF patients, the degranulation capability was measured by standard CD107 degranulation assay. Interestingly, the CD56^DIM^ NK-cells from IFN-OFF patients seemed to degranulate without stimulation, but there was no statistically significant difference when compared to the healthy controls (CD107 expressing cells in IFN-OFF group median 6.5% vs. 3.8% in healthy, p = 0.11, Figure 7A). After the stimulation with K562 cells, the degranulation was similar in IFN-OFF patients as in healthy controls (median IFN-OFF 12.9%, healthy 17.5%, p = 0.28, Figure 7B). Similarly, the cytokine production (TNF-α/IFN-γ) after the K562 stimulation did not markedly differ between the groups, but overall, the NK-cells from IFN-OFF patients were poor cytokine producers (Figures 7C and 7D).

## Discussion

The re-introduction of IFN-α to CML treatment has gained significant interest lately. Several studies have shown that the combination of IFN-α with imatinib results in better and faster treatment responses [15], [21] The addition of IFN-α to imatinib treatment has also been reported to increase the possibility to discontinue treatment successfully. For example, Burchert et al. has reported that 75% of CML patients receiving IFN-α/imatinib combination therapy are able to stay in remission after discontinuing imatinib treatment [19], whereas a lower frequency (30–40%) of successful treatment discontinuations have been observed after imatinib monotherapy [22], [23]. IFN-α therapy has also been shown to be effective in some TKI resistant patients such as in difficult-to-treat T315I mutation positive patients [24], [25]. Whether the beneficial results of IFN-α therapy are due to immunological properties is under debate, but also our results support the view that successful IFN-α therapy induces numerical and functional changes in the immune effector cells, which may contribute to excellent treatment responses.

In this project we focused to study the immune cell function and phenotype. Our cohort consisted of IFN-α monotherapy treated CML patients, which is a very rare group of patients due to current use of TKIs as a standard first-line treatment. However, as the treatment responses can be considered exceptional in these patients (either state of the minimal residual disease with IFN-α monotherapy or long-lasting remission after IFN discontinuation), they may help us to understand the requirements for successful treatment discontinuation. Interestingly, our results showed that IFN-OFF patients had enlarged CD8^+^ central memory T-cell compartment. These cells are CCR7^+^CD45RA^−^ cells that have encountered their cognate antigen, expanded, and they act mainly as a reservoir in the lymph nodes for later effector functions against the same antigen. Both the CD8^+^ central and effector memory T-cells have been used in the adoptive cancer immunotherapy as they have high lytic capacity and are able to produce IFN-γ as also observed in our study [26], [27]. Especially antigen specific CD8^+^ central memory T-cells have shown to possess superior antitumor immunity in vivo and eradicate even large melanoma tumors [28]. Although the antigen specificity of CD8^+^ central memory T-cells is not known in our patients, the CD8^+^ central memory T-cell compartment was markedly different in IFN-OFF patients when compared to healthy controls, and it could be hypothesized that these cells have a role in anti-tumor immunity. Together with a low number of CD8^+^_TEMRA_ cells (recently activated peripheral effectors) observed in IFN-OFF group, the findings are in line with the stable remission status of the IFN-OFF patients. Furthermore, it is noteworthy that in the IFN-OFF group the median time without treatment was close to 4 years suggesting that the differences observed were no longer direct effects of IFN-α, but long-term changes in the immune system. This is supported with the data from the follow-up samples from some patients showing that the immune-profile is unchanged at the later time-points.

In line with our results, Usuki et al has demonstrated in a small cohort of CML patients (n = 9) that patients who were able to stay in remission after imatinib discontinuation had increased amount of CD45RO^+^ memory CD8^+^ cells when compared to healthy volunteers or relapsing patients [29]. This needs to be confirmed in larger patient cohorts, but it suggests that CML patients who are able to maintain the remission after the therapy discontinuation may share similar features in their immunoprofile despite of the pre-existing therapy.

In addition to changes in the CD8^+^ T-cell pool, IFN-OFF patients had significantly increased amount of CD4^+^ effector memory cells. CD4^+^ effector memory cells are able to respond rapidly to antigen encounter by cytokine secretion [30]. In accordance, our results showed that when MNCs from IFN-OFF patients were stimulated, CD4^+^ cells responded significantly more active by secreting Th1-type cytokines TNF-α/IFN-γ than CD4^+^ T-cells from healthy controls, and effector and central memory CD4+ cells were the main cytokine producers. The cytokine production in turn can activate the generation of cytolytic CD8^+^ T-cells [31] or act directly against the cancer [32], and thus, CD4^+^ effector memory cells are crucial for the generation of antitumor immune responses [33].

Similarly as shown in our previous report [20], we noticed that IFN-OFF group had an expansion of mature CD56^DIM^CD62L^LOW^CD27^LOW^CD57^+^ NK-cells in the peripheral blood. Although CD56^DIM^ NK-cells have typically been suggested to act as cytotoxic cells, to our surprise, the killing activity in response to K562 cells was lower in IFN-OFF patients than in healthy controls. This is somewhat in contrast to previous results, which have shown that during IFN-α therapy, NK-cell cytotoxicity against K562 cells and autologous CML cells is increased [34], [35]. However, those results were obtained from patients who had just started IFN-α therapy compared to our IFN-OFF group, which has already been without treatment for several years. Constant activation with IFN-α can also lead to changes in the NK-cell phenotype [36], and the CD57 expression found in most of the NK-cells in IFN-OFF group also implies to exhausted terminal stage cells. This could explain why they did not react properly against third party target cells (K562) *in vitro*. Supporting this theory, it has been reported that CD56^DIM^CD62L^−^ NK-cells are not as capable of proliferating as CD56^DIM^CD62L^+^ NK-cells [37].

In addition to direct cytolytic abilities, NK-cells may also have other unknown functions in tumor immunology such as memory properties. The participation of NK-cells in the adaptive immune system is still under debate, but recent studies suggest that NK-cells are able to live long [38] and produce memory-like responses [39]–[41]. However, there is no specific immunophenotype known for NK memory cells [42], and therefore we were not able to analyze this aspect in our patient cohort.

Taken together, our results show that IFN-OFF patients have significant numerical and functional changes in both the T- and NK-cell compartments, which may contribute to the maintenance of prolonged remission after successful IFN-α discontinuation. Further studies in upcoming clinical trials evaluating the effect of IFN-α+TKI combination are needed to confirm these preliminary results.
